# Novel Application of Immersive Virtual Reality Simulation Training: A Case Report

**DOI:** 10.5435/JAAOSGlobal-D-21-00114

**Published:** 2021-11-18

**Authors:** Ryan Lohre, Lise Leveille, Danny P. Goel

**Affiliations:** From the Department of Orthopaedics, University of British Columbia, Vancouver, Canada.

## Abstract

**Case:**

A percutaneous pinning of a slipped capital femoral epiphysis is described after the use of immersive virtual reality (iVR) training. This case report documents the first reported example of an immediate translation of surgical skill from iVR to the operating room.

**Conclusion:**

There is increasing evidence for the use of iVR in orthopaedic education. Several randomized controlled trials demonstrate improved trainee performance relative to control when measured in analogous operating room assessments. This is the first case report demonstrating direct patient care after the use of iVR. The implications of cost-effectiveness through skill transfer and patient safety are highlighted.

Slipped capital femoral epiphysis (SCFE) is a common hip disorder affecting children, with incidence varying by geographic location.^1^ Classification of SCFE is based on history, physical examination, and radiographic features.^2^ Symptom temporality defines acute, acute-on-chronic, or chronic SCFE, whereas symptom severity determines ambulation and SCFE stability.^2^ A SCFE is radiographically classified using frog-leg lateral images by the Southwick angle as mild (<30°), moderate (30 to 50°), or severe (>50°).^3^ Stability of the SCFE by inability to weight-bearing informs surgical treatment options. Percutaneous fixation in situ remains the benchmark treatment for stable SCFE.^4^ Unstable SCFE treatment has been described as closed reduction (serendipitous or through gentle traction) and percutaneous fixation, open reduction with decompression and fixation, or open reduction with surgical dislocation and subcapital corrective realignment (modified Dunn procedure).^5^ Aligning more severe (>50°), unstable SCFE to preslip anatomy may reduce slip progression after fixation.^6^ In situ fixation predisposes to femoroacetabular impingement; however, open reduction procedures increase the risk of osteonecrosis and chondrolysis.^5^

When performing percutaneous in situ pinning, it is important for the surgeon to recognize that the metaphysis translates and angulates anterolaterally and that the main blood supply to the epiphyseal segment lies posterosuperiorly, as supplied by the lateral epiphyseal vessels.^7^ Providing perpendicular fixation across the physis and avoiding perforating vessels means a starting point anterolateral on the femur as the slip progresses and lateral to the intertrochanteric line.^8^ It is well recognized that multiple attempts at wire or screw placements increase postoperative fracture risk.^2^

Immersive virtual reality (iVR) simulators in orthopaedic education have been shown to be valid, efficient, and a cost-effective means of acquiring technical and nontechnical skills.^9,10,11,12,13,14^ Studies have characterized improved skills in medical students and surgical trainees of varying years when doing fracture fixation and arthroplasty. These simulator systems allow users to practice virtual surgeries in a scaled, interactive environment through computer hardware interfaces including head-mounted displays and hand-based controllers that provide sense of touch feedback. Evidence of translation of acquired skill remains limited to surrogate operating rooms using cadavers or models.

We describe the first reported case and evidence of immediate skill acquisition and translation to a real operative environment by a PGY-5 orthopaedic trainee having trained in iVR to revise a failed percutaneous pinning of a SCFE.

The patient and parents provided informed consent after discussion that data would be submitted for publication.

## Case Report

**Figure 1:**
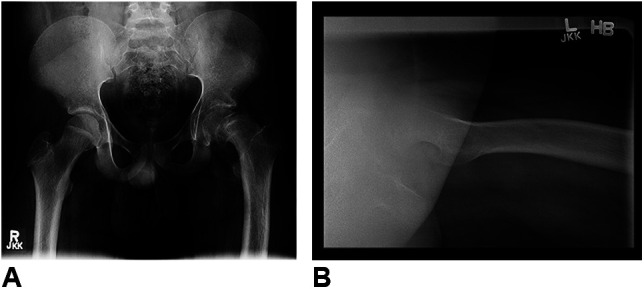
Initial radiographic investigations demonstrating a unilateral, left-sided slipped capital femoral epiphysis.

A 15-year-old, 99 kg, and 163 cm (body mass index 37.3) adolescent boy presented to an orthopaedic practice with complaints of chronic left groin and thigh pain for 6 months. He described atraumatic, nonprogressive pain with unilateral altered gait on weight-bearing. Anterior-posterior (AP) and cross-table lateral radiographs of the patient's pelvis and left hip were obtained, and he was diagnosed with a unilateral, idiopathic, stable, chronic SCFE. Figure 1 provides preoperative patient radiographs.

**Figure 2:**
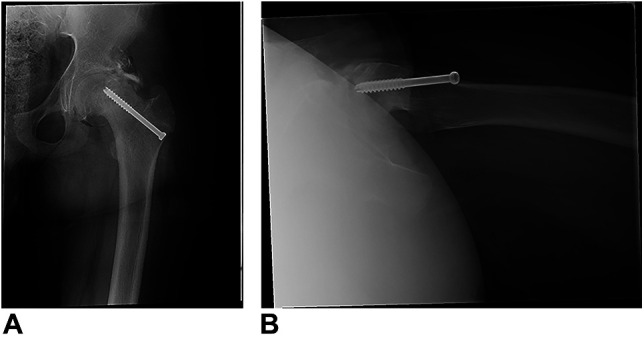
Postoperative images showing a single, partially threaded cancellous screw that does not capture the epiphysis of this severe slipped capital femoral epiphysis.

The patient and family were counseled on surgical management by the initial treating surgeon and brought to the operating room (OR) for a percutaneous pinning procedure. The surgeon was unable to complete the procedure, noting difficulty with visualization and interpretation under fluoroscopy to obtain the appropriate starting point and final screw placement. Intraoperative fluoroscopy amounted to 54.32 mGy cumulative radiation dose for the index procedure. The patient, after postoperative radiographs showing inadequate physeal fixation, was immediately returned to the operating room by the initial surgeon for hardware removal and referred to a pediatric center for management. Figure 2 demonstrates the initial fixation attempt.

**Figure 3:**
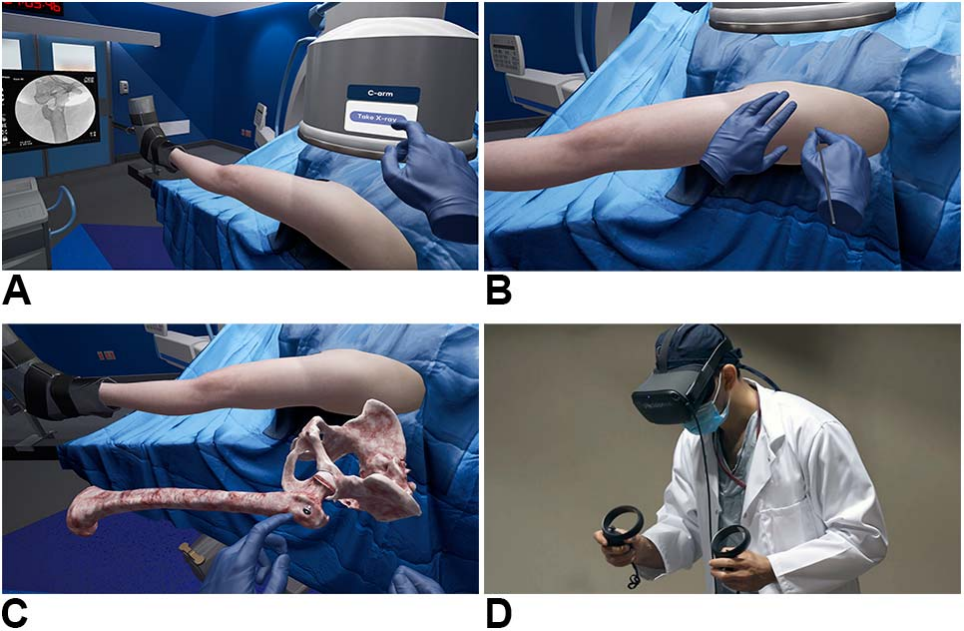
**A**, Image showing that simulated fluoroscopy allows the user to practice 3D localization for guidewire placement similar to a real OR. **B**, The PrecisionOS system allows for a haptic-controlled virtual surgery, not only providing visual and auditory cues but also differentiating soft tissue and bone by sense of touch (**C**) The iVR system allows for visuospatial understanding and direct interaction with patient anatomy. The screw is placed on the anterolateral femur, lateral to the intertrochanteric line to accommodate the slip angle. Full degrees of freedom are permitted while metrics on performance and implant position are observed. **D**, A surgical trainee using the PrecisionOS iVR system, shown with head-mounted display and position-tracking controllers. iVR = immersive virtual reality.

Preoperative planning was done at the pediatric center with repeat radiographs and CT scan. A single screw tract was visualized. Before the OR, the PGY-5 orthopaedic surgery resident on service did a SCFE training module four consecutive times using iVR (PrecisionOS Technology). The module provided a representative severe SCFE that the resident was able to actively percutaneously pin using a partially threaded cancellous screw in a virtual environment, complete with guide pin insertion, and fluoroscopic localization on interactive 3D anatomy. Figure 3 demonstrates the SCFE iVR environment using the PrecisionOS system.

**Figure 4:**
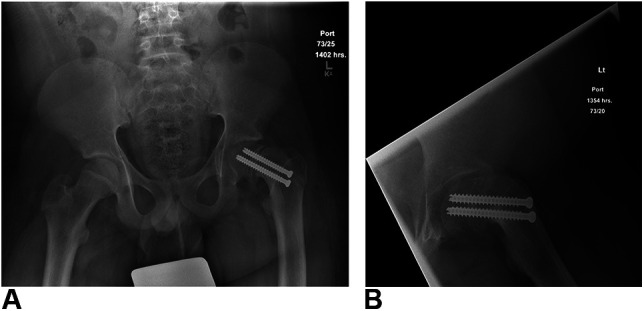
**A**, Anterior-posterior pelvis and (**B**) left hip lateral postoperative radiographs showing two screws traversing the physis of the severe SCFE with screw placement on the anterolateral femur to accommodate the 3D morphology of the SCFE. SCFE = slipped capital femoral epiphysis.

**Figure 5:**
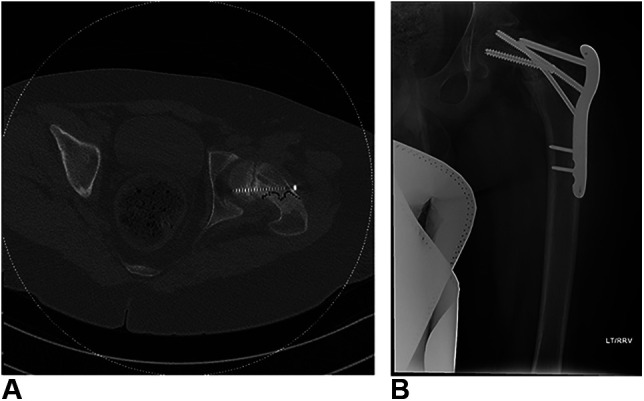
**A**, Preoperative CT scan demonstrating fracture propagation adjacent to erroneously placed screw tract from index procedure (**B**) Anterior-posterior radiograph of the revision open reduction and internal fixation

After iVR training, the patient was returned to the OR. The actual patient was placed supine on a radiolucent table with the left leg free-draped to obtain dynamic anterior-posterior and frog-leg lateral radiographs given the stable, chronic slip and reported difficulty with initial fracture table fluoroscopic visualization. With the presence of a pediatric fellowship trained orthopaedic attending surgeon, the senior resident independently placed two 7.3-mm cannulated screws perpendicular to the physis and lateral to the intertrochanteric line. Two screws were used given the severity of SCFE and patient size. Screw orientation and lengths were carefully considered to maximize fixation and prevent impingement. A single attempt was undertaken for Kirschner wire and screw placement to minimize cortical perforation given the previous surgery. Cumulative radiation dose by fluoroscopic imaging amounted to 7.13 mGy. Postoperative radiographs demonstrate appropriate screw orientation and depth, with greater than five threads across the physis. Figure 4 demonstrates this fixation. The patient was discharged from hospital when deemed safe to ambulate on postoperative day 3. Unfortunately, the patient fell while out of hospital one week after discharge and sustained a left-sided periprosthetic, pertrochanteric hip fracture. After readmission to the children's hospital, a CT scan was obtained and the patient underwent an open reduction and internal fixation using a proximal femoral locking plate. At 3-month follow-up, the patient is ambulating independently without pain and demonstrates radiographic union although there has been some interval fracture subsidence and there is a risk of screw perforation. Figure 5 provides a representative preoperative CT scan image and postoperative radiographs of the revision hip fixation.

## Discussion

The fundamental principles of percutaneous fixation for SCFE are dependent on regional anatomy, with screw fixation dependent on anterolateral cortical starting point and slip progression. Aronsson et al.^2^ illustrate that appropriate visualization of the physis must occur before pinning is attempted. Similarly, they describe a single attempt at wire localization because multiple attempts increase the rate of postoperative fracture. Severe SCFE in adolescents may be infrequently encountered by nonpediatric subspecialty orthopaedic surgeons, and as such, skill decay in conceptualization of the distorted anatomy and subsequent surgical fixation may occur. Percutaneous pinning of a severe slip requires a different start point and trajectory when compared with the more commonly done percutaneous pinning of femoral neck fractures to successfully achieve adequate fixation. In this case, failure of visualization and conceptualization resulted in complication and revision surgery.

In this case report, the PGY-5 had not done a SCFE procedure in the 2 years leading up to the reported case. Previous case volume was three mild SCFE percutaneous fixations. The iVR simulator allowed for effective refresher training through immersive learning with individualized performance feedback and productive failure in a safe environment. The PGY-5, when using the PrecisionOS software before surgery, sequentially improved their Precision Score (virtual performance metric) when practicing on a severe SCFE model from 70% to 93% (maximum score 100%). This score accounts for a unique combination of guidewire attempts, screw placement, erroneous placement including starting position and screw depth, fluoroscopy use, and overall procedural time. The Precision Score has been validated as a reliable performance metric when training in shoulder arthroplasty.^15^ The duration of case performance in iVR was reduced from 8 minutes 44 seconds to 3 minutes 46 seconds, equating to a 232% reduction in training time. Virtual fluoroscopic images were reduced from 42 to 5, a reduction of 840%. The virtual pelvis and femur are removable from the virtual patient and can be closely examined, rotated, and placed back through the virtual skin, assisting in understanding the more complex three-dimensional anatomy of the severe SCFE and start point relative to external anatomy. After these iVR sessions, the PGY-5 read information on SCFE pathophysiology for improved understanding in preparation for surgery. On the day of OR, two screws were placed by the PGY-5 in appropriate start point, trajectory and depth across the femoral physis. Approximately 7.6× less cumulative radiation dose was used compared with the index procedure. A postoperative radiograph (Figure 4) shows successful position of the revision in situ pin position.

Despite the evidence of iVR use, recognition and understanding of availability by the surgical community likely remains limited. Use of this simulation technology by surgeons may improve both cognitive understanding and psychomotor skills for infrequently done procedures. Alternatively, the system allows for multiplayer experiences. The initial treating surgeon may have done a procedure rapidly in iVR with a pediatric consultant orthopaedic surgeon remotely in virtual consultation to refresh specific knowledge and skill before surgery. For trainees, iVR simulators can provide effective and efficient adjunctive, supplemental training to traditional learning formats and operative experiences.

**Figure 6:**
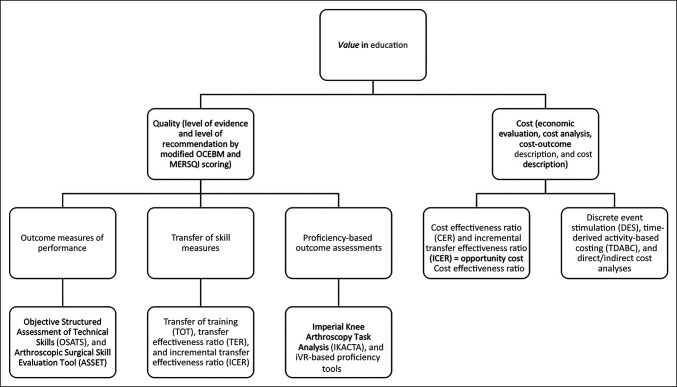
A flow diagram of the Porter and Teisberg^17^ concept of value applied to orthopaedic surgical education. Evidence-based measures of quality and cost are provided as examples for study. MERSQI = Medical Education Research Quality Instrument.

Theoretically, iVR has unique cost-effectiveness compared with traditional simulation formats in that it is portable, has cost conscious hardware, and can be readily updated. Immersive virtual reality has been shown to be 34× more cost-effective in training than a traditional course format.^15^ Considering our representative case, by improving initial surgical management through incorporation of refresher training using iVR simulation, notable reductions in direct costs may have been achieved by reducing multiple operations, hospital stay, and medical transfers. If we consider opportunity cost is equal to the difference of returns for most profitable investment choice and what is actually chosen, then continuing with traditional training structures and practice patterns in the absence of iVR may present missed opportunity costs for trainees, surgeons, and health care organizations alike. Figure 6 demonstrates a proposed value framework of quality and cost considerations in orthopaedic educational study based on Porter and Kaplan's value equation.^16^ By adhering to this framework, training programs and health care providers may provide clearer definitions of high-value, low-cost training solutions.
